# Reply to the letter: “Is the experience of chronic pain different in frail older adults?”

**DOI:** 10.1055/s-0046-1822634

**Published:** 2026-06-09

**Authors:** Nayara Tasse de Oliveira Cirino, Marcos Paulo Miranda de Aquino, Camila Astolphi Lima, Fânia Cristina dos Santos, Mauricio de Miranda Ventura, Monica Rodrigues Perracini

**Affiliations:** 1 Universidade Cidade de São Paulo, Programa de Mestrado e Doutorado em Fisioterapia, São Paulo SP, Brazil.; 2 Universidade Federal de São Paulo, Escola Paulista de Medicina, Departamento de Medicina, Disciplina de Geriatria e Gerontologia, São Paulo SP, Brazil.; 3 Hospital do Servidor Público Estadual, Ambulatório de Geriatria, São Paulo SP, Brazil.

Dear Editor,

We would like thank Dr. Sah and Dr. Kumbhalwar for their thoughtful comments on our article “Is the experience of chronic pain different in frail older adults?” and for recognizing the contribution of our study to the understanding of chronic pain in frail older people. We agree that untreated chronic pain in this population is a serious concern that is linked to complications such as poor sleep, social isolation, functional decline, and increased fall risk. 1

Regarding the first point on defining frailty, we agree that the Fried phenotype focuses on physical frailty, which may partly overlap with pain-related physical impairment and mobility limitations, particularly in cross-sectional studies. It is widely recognized that frailty and chronic pain share several biological, clinical, and psychosocial markers, explaining their frequent coexistence and mutual reinforcement in older adults. We selected the Fried criteria because of their widespread use and strong prognostic validity, enabling comparison with previous research on pain and frailty. However, we acknowledge that phenotype-based frailty could overestimate associations driven by shared markers of physical functioning, and that a cross-sectional design limits the understanding of the frailty-pain cycle. Future longitudinal studies should explore deficit-accumulation methods to better determine whether chronic pain leads to frailty, share common pathways, or both.

On the second point, regarding the prevalence of neuropathic pain and the use of the Douleur Neuropathique 4 (DN4), we emphasise that the DN4 is a highly-useful screening tool for neuropathic pain, and it was employed as a validated instrument. 2 We used the widely-recommended cut-off of ≥ 4 to suggest *probable* neuropathic pain, but not as a substitute for a full neurological diagnosis. Therefore, in our study, it was used as a screening instrument. We agree that many older adults present with mixed nociceptive–neuropathic pain, and that questionnaire-based classification alone can overestimate pure neuropathic pain, especially in frail subgroups. Among frail individuals, neuropathic pain should always be assessed and managed, whether neuropathic or mixed in nature. Accordingly, we described our findings as positive DN4 screens rather than definitive diagnoses of neuropathy, and we emphasized that treatment decisions—such as initiating any therapeutic interventions—should be based on thorough clinical assessment, taking into account comorbidities, functional status, and susceptibility to side effects. Ideally, a neurological examination should be performed when considering neuropathy-targeted treatments. We agree that initiating anticonvulsants or serotonin–norepinephrine reuptake inhibitors should never be based solely on questionnaire scores. Clinical assessment and personalized pain-management strategies are essential, rather than the indiscriminate prescription of medications. 3

Concerning the third point, on the analytical handling of psychological burden, we appreciate the emphasis on pain-induced depression as a potentially-mediating pathway. As stated in our methods, the primary aim of the study was to describe differences in pain, neuropathic features, and pain-related depression across frailty strata rather than to test causal or mediational models. The mediation analysis regarding chronic pain, pain-induced depressive symptoms (as captured by the GEAP-b - Geriatric Emotional Assessment of Pain (Brazilian version)), and frailty would indeed provide mechanistic insight, and we agree that such modelling could better clarify whether depression operates as a key driver of functional decline, a central objective for planned longitudinal extensions of our cohort.

We are grateful for the correspondents' recognition of the need for frailty-aware pain assessment and multidisciplinary management in geriatric populations, a need strongly supported by our findings. Their comments highlight important avenues for refinement—particularly the use of alternative frailty constructs, the incorporation of confirmatory neurological evaluation for neuropathic pain, and formal mediation analyses of psychological distress—that will inform the design of our future studies.
